# Development and Characterization of Magnetic SARS-CoV-2 Peptide-Imprinted Polymers

**DOI:** 10.3390/nano11112985

**Published:** 2021-11-06

**Authors:** Beatriz Fresco-Cala, Soumya Rajpal, Tamara Rudolf, Benedikt Keitel, Rüdiger Groß, Jan Münch, Alex D. Batista, Boris Mizaikoff

**Affiliations:** 1 Institute of Analytical and Bioanalytical Chemistry, Ulm University, 89081 Ulm, Germany; rajpalsoumya@yahoo.com (S.R.); tamara.rudolf@uni-ulm.de (T.R.); benedikt.keitel@uni-ulm.de (B.K.); boris.mizaikoff@uni-ulm.de (B.M.); 2 Department of Biochemical Engineering and Biotechnology, Indian Institute of Technology Delhi, New Delhi 110016, India; 3 Institute of Molecular Virology, Ulm University Medical Center, 89081 Ulm, Germany; ruediger.gross@uni-ulm.de (R.G.); jan.muench@uni-ulm.de (J.M.); 4 Hahn-Schickard Institute for Microanalysis Systems, 89077 Ulm, Germany

**Keywords:** dopamine, molecularly imprinted polymers, magnetic imprinted particles, peptide imprinting, epitope imprinting, surrogate imprinting, fragment imprinting, SARS-CoV-2, virus imprinting

## Abstract

The development of new methods for the rapid, sensitive, and selective detection of SARS-CoV-2 is a key factor in overcoming the global pandemic that we have been facing for over a year. In this work, we focused on the preparation of magnetic molecularly imprinted polymers (MMIPs) based on the self-polymerization of dopamine at the surface of magnetic nanoparticles (MNPs). Instead of using the whole SARS-CoV-2 virion as a template, a peptide of the viral spike protein, which is present at the viral surface, was innovatively used for the imprinting step. Thus, problems associated with the infectious nature of the virus along with its potential instability when used as a template and under the polymerization conditions were avoided. Dopamine was selected as a functional monomer following a rational computational screening approach that revealed not only a high binding energy of the dopamine–peptide complex but also multi-point interactions across the entire peptide template surface as opposed to other monomers with similar binding affinity. Moreover, variables affecting the imprinting efficiency including polymerization time and amount of peptide and dopamine were experimentally evaluated. Finally, the selectivity of the prepared MMIPs vs. other peptide sequences (i.e., from Zika virus) was evaluated, demonstrating that the developed MMIPs were only specific for the target SARS-CoV-2 peptide.

## 1. Introduction

The ongoing COVID-19 pandemic has affected a large percentage of the world’s population, including collapsing hospitals and medical services in almost every country [1,2]. Patients infected with COVID-19 can be asymptomatic or present mild symptoms such as coughing or fever and medical complications such as pneumonia and kidney injury [3,4,5]. Moreover, the ease of transmission of this virus has made its containment difficult, forcing the governments to limit social life [6]. One of the key ways to control infections is the conducting of tests that rapidly determine whether a patient is infected with SARS-CoV-2. Therefore, efforts have been geared towards the development of fast and cheap methodologies that allow for the detection of this coronavirus with high sensitivity and selectivity [7,8,9,10,11].

Molecularly imprinted polymers (MIPs) are excellent candidates for assay-associated molecular recognition schemes due to the formation of specific recognition sites in their polymeric structure and their capability to selectively bind to target compounds (e.g., viruses) [12]. In this way, MIPs are currently an attractive alternative to existing conventional techniques for detecting viruses (e.g., microscopy analysis and antibody affinity tests) because they can be prepared to quickly, easily, cheaply, and selectively extract one or more types of target viruses from complex samples such as biological fluids. In addition, these synthetic materials have other additional advantages such as high stability and robustness towards most organic solvents in a wide pH range, as well as resistance against high temperatures and pressures [13,14,15]. Although there is a wide range of synthetic routes to prepare MIPs in terms of the types of polymerization and monomers, as well as the morphology and size of the desired final solids, the addition of the template to the polymerization mixture is required in all cases. Thus, due to the polymerization reaction being carried out in the presence of the template molecule, the selection of the polymerization method, including the reaction conditions such as temperature and solvents, is crucial because these variables strongly affect not only the morphology and size of the final polymer but also the imprinting effect. The template can be the target (bio)molecule itself, a part or fragment of the target, or even another structurally and chemically similar compound (i.e., surrogate) [16]. Considering the experimental problems associated with the use of infectious biological species such as viruses as the template during the polymerization step, fragment imprinting is one of the most interesting and attractive strategies. In this case, for the preparation of MIPs with selective recognition toward a target virus, a protein or a peptide (i.e., a fragment of a relevant protein) may be used as a template instead of the real virus. Another vital variable that must be considered in a successful imprinting process (i.e., the creation of selective binding sites within the polymer matrix) is the selection of appropriate functional monomers. The monomers used for the preparation of MIPs must have functional groups that may interact (non-covalently) with the template. Hence, computational screening is an efficient tool for aiding in the selection the most adequate functional monomer(s) from a wide range of existing molecules. The rational screening of monomers using molecular mechanics is commonly used to predict MIP performance based on the selection of the highest binding constant of a potential monomer–template complex. Specifically, the strength of the monomer–template interaction in a pre-polymerization complex can be estimated by mapping the interactions of monomers at multiple residues of the protein/peptide. In a previous study by Boroznjak et al. [17], a molecular docking approach was used to select suitable functional monomers for imprinting immunoglobulin G (IgG). The docking of phenylenediamine (mPD), dopamine, and 3,4-ethylenedioxythiophene with the template revealed similar binding scores. However, mPD was involved in multi-point interactions at the fragment antigen-binding (FAB), fragment crystallizable (Fc), and hinge regions of IgG. A more uniform arrangement around IgG was thus proposed to enable more selective binding moieties and experimentally confirmed as the best performing monomer. To date, monomer screening is based on the scoring functions of software packages such as Autodock, Vina, and Glide, which can reliably predict the binding energy of functional monomers with macromolecular proteins and peptides [18,19].

In the present study, magnetic peptide-imprinted polymers (MMIPs) were developed for the detection of SARS-CoV-2. Magnetic nanoparticles (MNPs) were synthesized, and then a layer of polydopamine (PDA) was formed via the self-polymerization of dopamine at pH = 8.5. The selected SARS-CoV-2 peptide (template) was derived from the receptor-binding domain of the viral spike protein and used as a template during the polymerization. Dopamine was selected as the optimum functional monomer for the formation of selective binding moieties for the target peptide among a list of 19 commonly used monomers based on molecular docking studies obtained via Autodock Vina. Moreover, variables affecting the imprinting efficiency, including time of the polymerization reaction and amount of peptide and dopamine, were experimentally evaluated. Finally, a selectivity study was performed by testing the developed MMIPs against another peptide sequence derived from the Zika virus glycoprotein.

## 2. Experimental Section

### 2.1. Reagents and Materials

The chemicals used for the synthesis of MNPs were iron (II) chloride (FeCl_2_·4H_2_O) and iron (III) chloride (FeCl_3_·6H_2_O), and both of them were acquired from Sigma-Aldrich. Dopamine was purchased from VWR. A Tris buffer (20 mM; pH = 8.5) was used to prepare the solutions involved in the polymerization, as well as to prepare the peptide standards at a concentration of 10 mg/mL. SARS-CoV-2 peptide (Table 1) was used for the preparation of the MMIPs, as well as in the rebinding tests. This specific sequence was selected as part of the receptor binding domain of the spike protein that actively interacts with the ACE2 receptor, as reported by Lan et al. [20]. The selected peptide is composed of residues with active interactions with the ACE2 human receptor, which was employed as the template during molecular imprinting, thus resulting in a synthetic receptor that mimicked the ACE2 receptor interactions with the SARS-CoV-2 spike protein (Figure S1).

The selectivity study was carried out using a peptide from Zika virus (Table 1). This sequence was selected because it is located in the external part of this virus, which is the specific part recognized by different antibodies [21]. Both peptides were purchased from GenScript (www.genscript.com, accessed on 4 November 2021).

### 2.2. Instrumentation

A JOEL JEM-1400 (TEM) was used for the characterization of the MNPs, MMIPs, and magnetic non-imprinted polymers (MNIPs). Therefore, a drop of an aqueous dispersion of these solids was placed on a copper grid and then dried to retain the particles on the grid.

UV/Vis spectroscopy (Specord S6000 from Analytik Jena, Germany) was used for the analysis of the solutions containing the peptides before and after the rebinding procedure. The measurements were performed at 280 nm in a Suprasil quartz glass cuvette with an optical path length of 2 mm (Hellma Analytics, Müllheim, Germany).

Infrared measurements were performed using a Bruker Alpha II FTIR spectrometer (Bruker Optics GmbH, Ettlingen, Germany) equipped with a platinum attenuated total reflection (ATR) accessory and a diamond ATR crystal. Spectra were collected between 4000 and 600 cm^−1^ at a spectral resolution of 1 cm^−1^, with 64 coadded scans each.

Dynamic light scattering (DLS) measurements of aqueous dispersions of the MNPs were performed with a Zetasizer NANO ZSP (Malvern, Herrenberg, Germany) after 5 min of sonication in an ultrasound bath. The measurement parameters were as follows: a laser wavelength of 632 nm (He–Ne), a scattering angle of 173°, a measurement temperature of 25 °C, a viscosity of 0.8872 mPa·s, and a dispersant refractive index of 1.330. The material refractive index was 2.0. A disposable polystyrene cuvette (minimum volume: 1 mL) was used for the measurements.

### 2.3. Computational Methods

The structural files for all monomers were downloaded from the PubChem databank (Table 2). The crystal structure of SARS-CoV-2 receptor-binding domain (PDB ID:6M0J) was processed using UCSF Chimera 1, and the peptide structure was extracted using the sequence selection tool (Figure S1). Autodock Vina—the most commonly employed open-source molecular docking software that is based on AMBER force field suitable with proteins, nucleic acids, and other organic molecules [22]—was employed for molecular docking. The ligand files were converted to pdbqt files after setting the torsional degrees of freedom based on the detected rotatable bonds. For the docking of monomers, the exhaustiveness value was set to 400 to improve the accuracy of prediction [23]. Using Autodock tools, polar hydrogens were added to the peptide, and water molecules were deleted. As each of these monomers (Table 2) requires different solvent and polymerization conditions, water was preferably deleted before docking. A grid box with dimensions of 62 × 98 × 66 Å was centered on the peptide. The ligand-binding energy is represented as kcal/mol. The largest ligand-binding affinity values were compared for each monomer. Furthermore, all docking clusters were used for comparison to analyze favorable multi-point interactions on the surface of the peptide. The BIOVA Discovery Studio Visualizer and UCSF Chimera were employed to analyze the interactions with each docking pose.

### 2.4. Synthesis of MNPs

MNPs were prepared using a co-precipitation method [24] that consisted of mixing 24 g of FeCl_3_·6H_2_O and 9.8 g of FeCl_2_·4H_2_O and then subsequently dissolving them in 100 mL of Milli-Q water. This solution was placed in an oven at 80 °C for 30 min in order to achieve a complete dissolution of the solids. Next, the solution was cooled at room temperature, and 50 mL of ammonia were added. Instantly, a brown magnetic precipitate was formed. The solid was separated with an external magnet, and then it was washed several times with Milli-Q water and methanol. Finally, MNPs were dried under vacuum at 40 °C.

### 2.5. Synthesis of Magnetic SARS-CoV-2 Peptide-Imprinted Polymers

A selective peptide-imprinted layer of dopamine was polymerized over MNPs. For that, a dispersion of the MNPs in a Tris buffer at a concentration of 40 mg/mL was prepared. We mixed 50 µL of this dispersion with 150 µL of a solution of the peptide (template) at 10 mg/mL in a Tris buffer. Then, 450 µL of a Tris buffer and 100 µL of a Tris buffer solution containing dopamine at 22.5 mg/mL were added to the mixture. The polymerization was carried out in protein LoBind tubes for 24 h shaking in a rocking shaker at 120 rpm. Once the polymerization reaction finished, the MMIPs were recovered with an external magnet and washed several times with ethanol and water until no peptide signal was obtained in the UV–vis. MNIPs were prepared following the same procedure in the absence of the peptide template. Instead of the template solution, the same volume of Tris buffer (150 µL) was added to the polymerization mixture to maintain the volumes and concentrations of the MNPs and dopamine.

### 2.6. Binding Studies

Two milligrams of the MMIPs/MNIPs were dispersed in 750 µL of an aqueous peptide solution at a concentration of 1 mg/mL. Next, this solution was incubated on a rocking shaker at 120 rpm for 3 h at room temperature. After separation with a magnet, the supernatant was withdrawn and analyzed by UV–vis (280 nm) to quantify the amount of SARS-CoV-2 peptide extracted by the MMIPs/MNIPs. The samples containing the Zika peptide were incubated with the MMIPs/MNIPs using the same procedure. However, ninhydrin was used to react with the Zika peptide and therefore generate a purple color. For that, 0.3 mL of supernatant was diluted with 0.2 mL of a PBS buffer. Next, 0.5 mL of a solution of 2% ninhydrin was added, and the mixture was vortexed for 3 s. This mixture was incubated for 17 min at 95 °C and 650 rpm. Finally, it was analyzed with UV–vis spectroscopy (570 nm).

The adsorption capacity (Q, mg/g) of the MMIPs and MNIPs towards the target peptide was calculated with the following equation:

Q = (C_0_ − C_t_)V/m

where Q (mg/g) is the adsorption amount, C_0_ (mg/mL) is the initial peptide concentration, C_t_ (mg/mL) is the peptide concentration in the supernatant after rebinding, V (mL) is the volume of the sample, and m (g) is the mass of the used MNPs.

The imprinting factor (IF) was used to evaluate the selectivity of the prepared MMIPs toward the SARS-CoV-2 peptide. The IF was calculated with the following formula:

IF = Q_MMIP_/Q_MNIP_

where Q_MMIP_ and Q_MNIP_ (mg/g) are the adsorption capacity of MMIPs and MNIPs toward the target peptide, respectively.

### 2.7. Selectivity Studies

MMIPs prepared with the selected SARS-CoV-2 peptide as a template and their corresponding MNIPs prepared without the presence of a template were used for the rebinding of a Zika peptide sequence. The rebinding procedure was carried out at the conditions described above (i.e., same conditions as those for SARS-CoV-2 peptide). Moreover, the experimental results for both peptides were compared with the predictions with computational methods. For that, peptides were extracted using UCSF Chimera from the protein crystal structure (PDB ID: 6CO8). Using Autodock tools, polar hydrogens were added to the peptide. A grid box with dimensions of 52 × 60 × 66 Å was centered on the peptide. Docking was performed with dopamine using the scoring functions of Autodock Vina.

## 3. Results and Discussion

### 3.1. Computational Screening

Initially, we screened suitable functional monomers from a library of commonly employed monomers for peptide templates (Table 2). This molecular screening using the docking software Autodock Vina enabled the quantitative evaluation of the monomer–peptide interactions including hydrogen bonding, van der Waals interaction, electrostatics, and hydrophobic forces.

**Figure 1:**
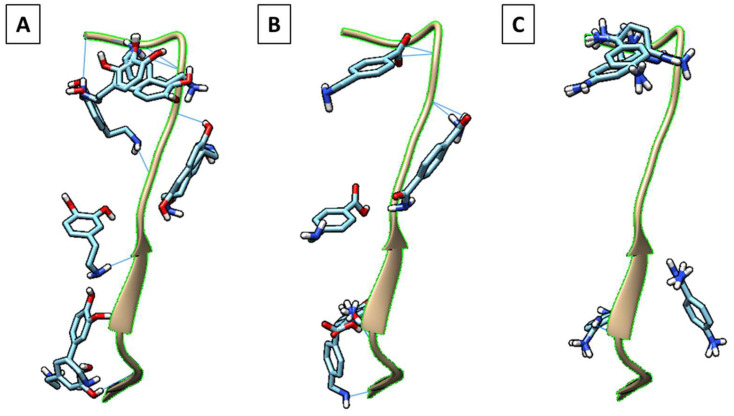
Binding poses of the ligands (**A**) 4-(aminomethyl)benzoic acid (MABA), (**B**) p-aminobenzamidine (PAB), and (**C**) dopamine clustered on the peptide that is in the center (highlighted with green). H-bonds are represented with solid lines. The monomer binding poses in each case were distributed in the binding energy range from −3.2 to −3.4 kcal/mol.

**Figure 2:**
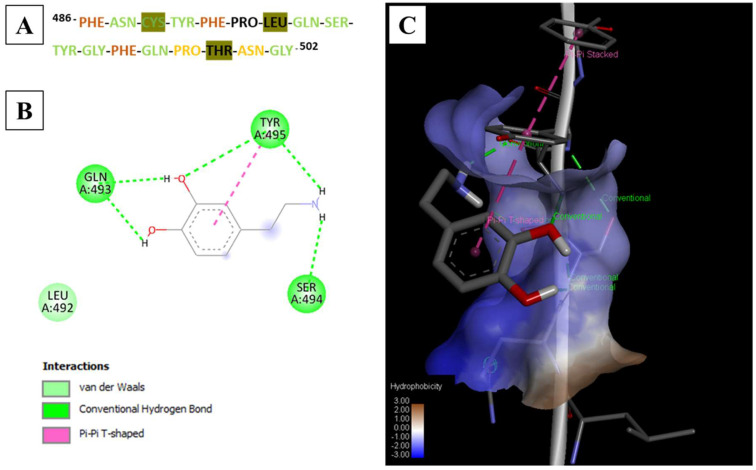
(**A**) Representation of types of interactions of dopamine with each amino acid residue (shown with one letter code) that are actively involved in H-bonding (green) and van der Waals interactions (black letters highlighted with green). When all the docking poses were visualized, residues were also shown to be involved in more than one type of binding interaction such as H-bonding with π–π interactions (brown) or van der Waal forces (yellow). The binding of most favorable docked pose with the peptide with a binding energy of −3.4 kcal/mol mapped in (**B**) 2D and (**C**) 3D, where green dotted lines represent hydrogen bonds, purple/pink areas represent π–π bond interactions, and blue areas represent solvent accessible surfaces.

The binding energies of nearly 20 monomers were distributed from −2.2 to −3.4 kcal/mol (Table 2). Among these, dopamine, 4-(aminomethyl)benzoic acid (MABA) (Figure 1A) and p-aminobenzamidine (PAB) (Figure 1B) were the highest scoring monomers, with a binding energy of −3.4 kcal/mol each. Based on the characteristic benzene ring in each of these monomers and the abundance of aromatic residues in the target peptide, the stacked π–π interactions in the monomer–peptide complex could be easily interpreted from the binding modes. Using UCSF Chimera, we analyzed the docked poses of the three monomers distributed across the energy range from −3.2 to −3.4 kcal/mol, and only dopamine was found to be uniformly bound over the entire SARS-CoV-2 peptide surface (Figure 1C). A typical antigen–antibody interface involves a group of interactions at multiple residues that results in a high-affinity complex; this should be effectively mimicked to maximize MMIP performance. Consequently, since the docked models of dopamine in the considered energy range interacted with almost all the amino acids of the peptide, dopamine was selected as the most suitable functional monomer for imprinting (Figure 1C). The different types of interactions were mapped across the peptide surface using Discovery studio, and a color-coded map was created to represent multi-point interactions (Figure 2A). This was based on the binding interactions analyzed in 2D and 3D for each docking outcome. An example with the most favorable binding mode is shown in Figure 2B,C. The already increasing importance of dopamine for protein imprinting complemented by our theoretical validation provided an impetus for the rational design of MMIPs for SARS-CoV-2 detection. Besides the hydrophobic interactions of dopamine with PHE and TYR residues (π–π), intermolecular interactions also include van der Waals forces mainly contributed by PRO, THR, and LEU residues. ASN, GLN, SER, PHE, and TYR residues are actively involved in H-bonding with dopamine. The presence of both a primary amine and two hydroxyl groups in dopamine enabled a very active network of H-bonds in the complex. The number of H-bonds formed were evidently highest in the complex with dopamine compared to the other monomers (Figure 1 and Table 2). The amino acids are also involved in other types of non-covalent interactions such as van der Waals, π–alkyl, and alkyl-based interactions that indicates strong complex formation, most favorable with dopamine compared to MABA and PAB. In this study, the choice of the monomer was based on an initial molecular mechanic (docking) study to select those monomers that could realize multi-point interactions over the entire peptide surface. Multiple docked complexes were analyzed both separately and all at once in order to closely mimic the molecular imprinting phenomenon, wherein several molecules (monomers) also interact variably over the entire peptide surface.

### 3.2. Synthesis and Characterization of Magnetic Epitope-Imprinted Polymers

MNPs were used as core materials to prepare magnetic epitope-imprinted polymers. They were prepared with a co-precipitation method [24]. Figure S2 shows the TEM micrographs of the prepared MNPs. As revealed in these photos, the MNPs were nearly spherical in shape and uniform in size with a diameter of ~10 nm. The size was also confirmed by DLS, obtaining a hydrodynamic average diameter of 45.57 nm, which was pretty similar to a previously reported value [24].

**Figure 3:**
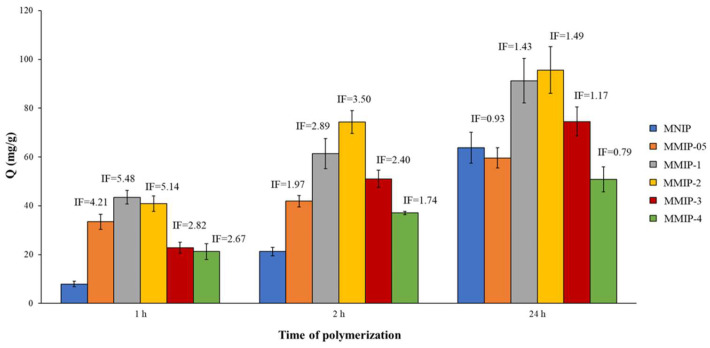
Evaluation of the binding capacity and imprinting factors (IFs) of the magnetic molecularly imprinted polymers/magnetic non-imprinted polymers (MMIPs/MNIPs) prepared with different amounts of SARS-CoV-2 peptide and different polymerization times.

Peptide-imprinted layers were obtained via the self-polymerization of the PDA onto the MNP surface at pH = 8.5 (20 mM Tris buffer solution) in the presence of the template (SARS-CoV-2 peptide). Initially, the concentration of dopamine in the polymerization mixture was fixed at 2 mg/mL, and the effect of the amount of peptide and the time of polymerization were studied. Table 3 lists the composition of the different polymerization mixtures of the prepared MMIPs/MNIPs. A SARS-CoV-2 peptide solution at 10 mg/mL in a Tris buffer was prepared, and different volumes (0–300 µL) were taken from it. Next, these volumes were added to the polymerization mixture. The effect of the time of polymerization was also evaluated, for which MMIPs/MNIPs polymerized for 1–24 h were tested. As can be seen in Figure 3, imprinting factors (IFs) between 2.67 and 5.48 were obtained for the MMIPs polymerized for 1 h. However, by increasing the polymerization time to 24 h, the obtained IFs were lower, although higher rebinding capacities (Q values) were obtained in all cases. This was due to the fact that the increase in polymerization time led to the formation of a greater amount of dopamine that increased the sorbent capacity of both MMIPs and MNIPs. Changes in the colors of the resulting polymers were also observed depending on their polymerization time (Figure S3). Longer times of polymerization gave rise to darker solids (at the beginning of polymerization, all mixtures had a yellowish-brown color; see Figure S4), which corresponds to the theory that compared to shorter polymerizations, a greater amount of PDA was formed (Figure S3).

Regarding the amount of SARS-CoV-2 peptide (template) added to the polymerization mixture, the intermediate amounts (MMIP-1 and MMIP-2) were the ones that achieved the best imprinting (IFs ranged from 5.14 to 5.48 for 1 h, from 3.89 3.50 for 2 h, and from 1.43 to 1.49 for 24 h); consequently, the highest Q values were obtained with them. Significant differences in the color of the solids prepared with different amounts of the SARS-CoV-2 peptide (and the same polymerization time) were also observed. As seen in Figure S3, the color was darker when the amount of peptide (template) was lower, while a lighter brown was found for polymerizations with higher amounts of peptide. Considering that a darker color indicated a greater formation of PDA (Figure S5), very high amounts of peptide (MMIP-3 and MMIP-4) affected the polymerization rate, forming less PDA. On the other hand, the polymers prepared without the peptide (MNIPs) or with very low amounts of it (MMIP-05) showed dark colors (the formation of PDA was successful), but they were little or not selective towards the SARS-CoV-2 peptide. Additionally, clear visual differences were observed in the dispersion of the MMIPs and MNIPs (Figure S4). The MNIPs resulted in aggregates a few seconds after the addition of the dopamine. These aggregates could be dispersed with a stronger shaking of the tubes, though they were formed again when the agitation stopped. On the contrary, the polymerization mixtures of the MMIPs (specially with high amount of peptide) remained completely dispersed. The interactions between the peptide and the dopamine probably avoided the instant aggregation of the molecules of the dopamine (and consequently of the MNPs). Thus, the addition of the peptide favored the strong dispersibility of the MNPs during the formation of the PDA-imprinted layer.

**Figure 4:**
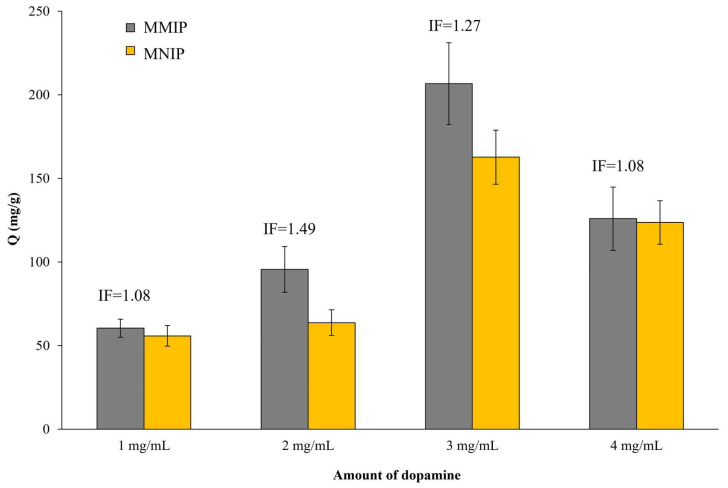
Evaluation of the binding capacities and IFs of MMIPs/MNIPs prepared with different amounts of dopamine.

Next, the concentration of dopamine during the polymerization was evaluated. For that, different polymerization mixtures were prepared with several amounts of dopamine (MMIP/MNIP-PDA1, MMIP/MNIP-PDA2, MMIP/MNIP-PDA3, and MMIP/MNIP-PDA4 were prepared with 1, 2, 3, and 4 mg/mL of dopamine, respectively). For this experiment, the amount of the peptide was set at 2 mg/mL and the polymerization reaction was carried out for 24 h (MMIPs with highest Q values). Figure 4 shows that the sorbent capacity of the MMIPs increased with the dopamine concentration up to 3 mg/mL (MMIP-PDA3), obtaining a Q value of 206.73 mg/g. However, the MNIP binding capacity also increased with the presence of PDA, which resulted in a lower IF at a dopamine concentration of 3 mg/mL than at 2 mg/mL. This result has been previously reported [25,26] due to the greater presence of functional groups (more amount of PDA in the final solid) that leads to a greater number of non-specific interactions. However, when the concentration of dopamine reach to 4 mg/mL, the binding capacities of both MMIP-PDA4 and MNIP-PDA4 decreased. This could have been due to the fact that a high amount of dopamine generated too much PDA, leaving the cavities or binding sites inside the polymeric structure (instead of on its surface). In this way, the cavities were not sufficiently accessible, with most of them not available for rebinding; the majority of interactions between the peptide and the PDA were non-selective (IF = 1.08).

**Figure 5:**
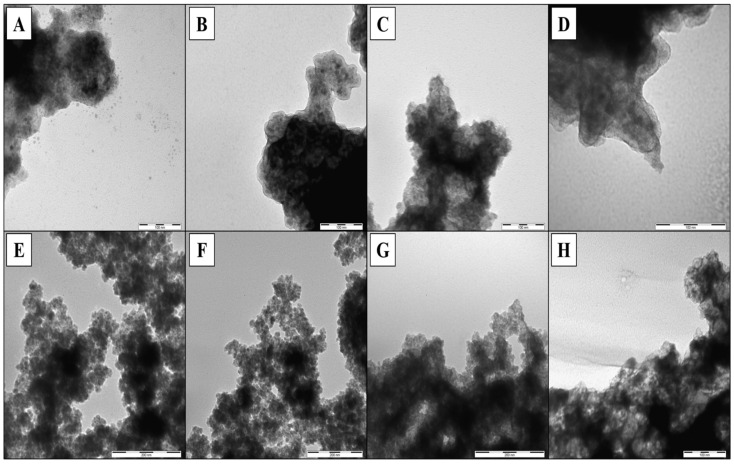
Transmission electron microscopy (TEM) images of MMIPs prepared with different concentrations of dopamine: (**A**) 1 mg/mL (MMIP-PDA1), (**B**) 2 mg/mL (MMIP-PDA2), (**C**) 3 mg/mL (MMIP-PDA3), and (**D**) 4 mg/mL (MMIP-PDA4); their corresponding MNIPs: (**E**) 1 mg/mL (MNIP-PDA1), (**F**) 2 mg/mL (MNIP-PDA2), (**G**) 3 mg/mL (MNIP-PDA3), and (**H**) 4 mg/mL (MNIP-PDA4).

**Figure 6:**
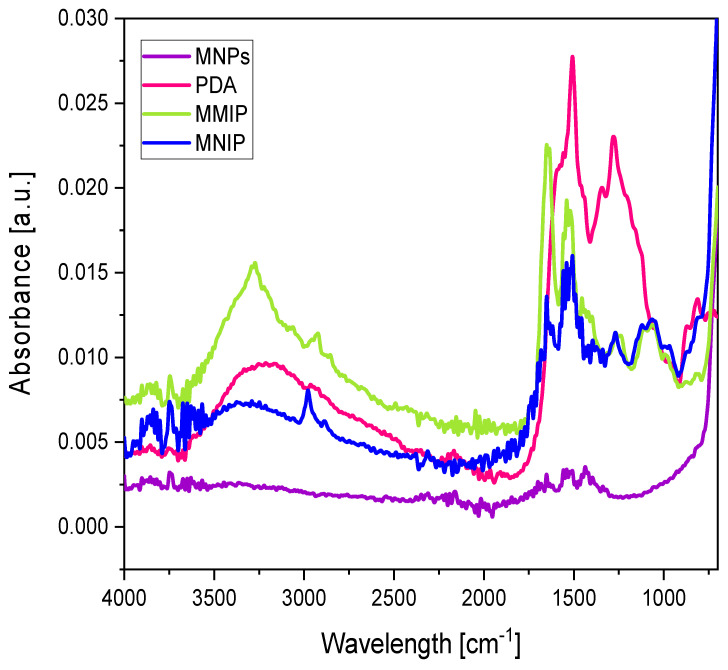
Fourier-transform infrared spectroscopy (FTIR) spectra of MNPs (**purple**), polydopamine (PDA) (**pink**), MMIPs (**green**), and MNIPs (**blue**).

The MMIPs/MNIPs prepared with different amounts of dopamine were characterized by TEM (Figure 5). The MMIPs and MNIPs looked very different compared to the MNPs without dopamine (Figure S2), but no significant differences were observed for the MMIPs in terms of the size or shape. However, TEM micrographs of MNIPs showed more granular polymers that could have been caused by the visual differences found between MMIPs/MNIPs (the formation of aggregates in NIPs during the polymerization step—Figure S4). The PDA, MNPs, MMIPs, and MNIPs were also characterized by FTIR (Figure 6). These spectra were used to verify the self-polymerization of dopamine on the surface of MNPs. It can be seen that MMIP/MNIP spectra presented the specific bands of MNPs, as well as representative peaks for the PDA. For instance, MNPs exhibited a characteristic and significant band at 580 cm^−1^ that could be assigned to the Fe–O–Fe vibration, and this band was also present in the MMIP and MNIP spectra. According to the PDA signals, a broad absorption band at 3700–3300 cm^−1^ and two characteristic bands of 1510 and 1333 cm^−1^ (ascribed to C = N and C–N–C stretching vibrations, respectively) were also found in the MMIP/MNIP spectrum [27,28].

Considering the results obtained with the different variables that affected the synthesis of MMIPs/MNIPs (i.e., polymerization time, amount of peptide and initial concentration of dopamine), we can conclude that an increase in PDA did not lead an increase in the IF in all cases. The obtained results showed that an increase in PDA generally resulted in a higher binding capacity (Q value). However, the diffusion of the peptide could have been negatively affected due to the greater difficulty for the peptide to access the binding sites that were not superficial. In this way, the binding capacities of the MMIP and MNIP were almost equal, giving rise to smaller IFs. For these reasons, intermediate but reasonably good Q and IF values, such as those obtained for MMIPs/MNIPs polymerized for 2 h (MMIP-2-2), would be optimal. In this case, the polymerization conditions led to MMIP-2-2 with a high Q value in addition to presenting a higher selectivity than the MNIP-2 (IF = 3.5).

### 3.3. Selectivity Studies

To evaluate the selectivity of MMIPs toward the SARS-CoV-2 peptide, a rebinding study was also carried out using a peptide sequence derived from the Zika virus glycoprotein (see Table 1). In this experiment, MMIPs/MNIPs that showed the best IFs (MMIP-1-1/MNIP-1, MMIP-2-2/MNIP-2, and MMIP-2-24/MNIP-24), as well as the MIP/NIP with the highest Q value (MMIP-PDA3/MNIP-PDA3), were used to rebind with the Zika peptide. As shown in Table 4, the Q values for the MMIPs/MNIPs prepared with 2 mg/mL of dopamine (MMIP-1-1/MNIP-1, MMIP-2-2/MNIP-2, and MMIP-2-24/MNIP-24) were similar since these polymers did not selectively bind the Zika peptide. Though a small increase in the sorbent capacity both the MMIPs and the MNIPs was observed with the increase of the polymerization time (especially between 1 and 2 h), the Q values and IFs for the Zika peptide were significantly lower than those obtained for the SARS-CoV-2 peptide. When the dopamine concentration was increased to 3 mg/mL (MMIP-PDA3/MNIP-PDA3), the Q values increased, which means that these solids showed a high binding affinity towards the Zika peptide. However, no significant difference was found between MMIP-PDA3 and MNIP-PDA3, which resulted in an IF of approximately 1, i.e., the Zika peptide was also not selectively bound to the MMIPs in this case.

To better understand these interactions, the Zika virus epitope was also docked with dopamine. The most favorable binding position showed higher binding energy values (−4.0 kcal/mol) than the SARS-CoV-2 peptide (−3.4 kcal/mol). However, the interactions in the first case were not observed across the entire macromolecule, similar to the case of SARS-CoV-2 peptide (Figure S6). Clearly, the set of interactions formed during the polymerization process—as also predicted from the docking studies—were unique to the SARS-CoV-2 spike peptide. The nearly equal binding capacities of the Zika peptide of both MMIPs and MNIPs also support the specificity towards the SARS-CoV-2 peptide.

## 4. Conclusions

MMIPs based on the self-polymerization of a dopamine layer coated on the surface of magnetic nanoparticles were prepared in the presence of a SARS-CoV-2 spike-derived peptide as the template. A computational screening approach was used to select the most suitable functional monomer, i.e., the one with the highest affinity towards the target peptide. Among the monomers studied here, three of them presented the highest scores, with a binding energy of −3.4 kcal/mol each. Hence, an in depth theoretical study analyzing the docked positions was executed, demonstrating that only dopamine uniformly binds across the entire peptide surface. Considering that its allows for rapid and biocompatible polymerization conditions, dopamine was also selected as the most suitable functional monomer. Non-imprinted control particles were also prepared following the same conditions without the addition of the template to the polymerization mixture. After the formation of the dopamine layer at the magnetic cores, both MIPs/NIPs preserved their magnetic properties, which facilitated the handling of these particles during the washing and binding steps. The synthesized MMIPs showed higher binding capacities than the corresponding MNIPs, which demonstrated that an imprinted layer of dopamine was coated the magnetic cores. Finally, the selectivity of the MMIPs was proven against another peptide (i.e., characteristic for Zika virus glycoprotein).

After observing these promising results, we believe that the MMIPs prepared in this work have great potential for use in the area of analytical chemistry since they allow for the selective recognition of the SARS-CoV-2 peptide. In this way, our efforts are now driven toward the selective detection of the complete SARS-CoV-2 in biological samples using these MMIPs, even in the presence of other viruses. Moreover, the obtained Q values lead us to think that with an appropriate elution of the retained viral particles and the use of an appropriate detection technique (e.g., high performance liquid chromatography coupled to a UV detector, HPLC–UV), low/realistic detection limits could be reached. In this way, the developed MMIPs could be used as a sensitive, selective, and cheap alternative to conventional methods.

## Figures and Tables

**Table 1:** Information about the selected peptides.

Peptide	Sequence (One Letter Code)	Sequence (Three Letter Code)	Source Protein	Sequence Start	Sequence End
SARS-CoV-2	FNCYFPLQSYGFQPTNG	Phe–Asn–Cys–Tyr–Phe–Pro–Leu–Gln–Ser–Tyr–Gly–Phe–Gln–Pro–Thr–Asn–Gly	PDB ID: 6M0J	486	502
Zika virus	MIVNDTGHETDENRA	Met–Ile–Val–Asn–Asp–Thr–Gly–His–Glu–Thr–Asp–Glu–Asn–Arg–Ala	PDB ID: 6CO8	151	165

**Table 2:** Virtual screening of a library of functional monomers for rationally predicting an effective molecularly imprinted polymers (MIP) design. A ranked list of docked monomers is arranged in ascending order of binding energy. Higher negative values (kcal/mol) indicate better binding affinity.

Monomers	Binding Energy (kcal/mol)
opamine	−3.4
4-(Aminomethyl)benzoic acid (MABA)	−3.4
p-aminobenzamidine (PAB)	−3.4
2-Acrylamido-2-methylpropane sulfonic acid	−3.2
Itaconic acid	−3.0
N-(3-aminopropyl) methacrylamide	−2.9
(Hydroxyethyl)methacrylate	−2.7
Aniline	−2.8
N-[3-(dimethylamino)-propyl]-methacrylamide	−2.8
N-isopropylacrylamide	−2.8
N-hydroxymethylacrylamide	−2.7
N-tert-butylacrylamide	−2.7
2-(Dimethylamino)ethyl methacrylate	−2.5
2-hydroxyethyl acrylate	−2.5
Glycidyl methacrylate	−2.5
Methacrylic acid	−2.5
Acrylamide	−2.2
Acrylic acid	−2.2
Methyl methacrylate	−2.3

**Table 3:** List of prepared magnetic molecularly imprinted polymers/magnetic non-imprinted polymers (MMIPs/MNIPs) and their respective polymerization conditions.

MMIPs/MNIPs	Amount of MNPs ^a^ (µL)	Amount of Peptide ^b^ (µL)	Amount of Tris Buffer (µL)	Amount of Dopamine ^c^ (µL)	Time of Polymerization
MNIP-1	50	0	600	100	1
MNIP-2					2
MNIP-24					24
MMIP-05-1	50	37.5	562.5	100	1 2 24
MMIP-05-2					
MMIP-05-24					
MMIP-1-1	50	75	525	100	1
MMIP-1-2					2
MMIP-1-24					24
MMIP-2-1	50	150	450	100	1
MMIP-2-2					2
MMIP-2-24					24
MMIP-3-1	50	225	375	100	1
MMIP-3-2					2
MMIP-3-24					24
MMIP-4-1	50	300	300	100	1
MMIP-4-2					2
MMIP-4-24					24

^a^ This volume was taken from a dispersion of magnetic nanoparticles (MNPs) at 40 mg/mL in a Tris buffer. ^b^ This volume was taken from a peptide solution at 10 mg/mL in a Tris buffer. ^c^ This volume was taken from a dopamine solution at 15 mg/mL in a Tris buffer.

**Table 4:** MMIP/MNIP selectivity study using SARS-CoV-2 and Zika peptides.

		Q (mg/g) ± SD (mg/g)				IF			
		MMIP-1-1/MNIP-1	MMIP-2-2/MNIP-2	MMIP-2-24/MNIP-24	MMIP-PDA3/MNIP-PDA3	MMIP-1-1	MMIP-2-2	MMIP-2-24	MMIP-PDA3
Zika	MMIP	14.02 ± 1.02	18.28 ± 1.04	18.67 ± 4.36	191.55 ± 22.98	0.89	0.72	0.82	1.08
	MNIP	15.69 ± 2.08	25.35 ± 1.93	22.68 ± 1.02	176.25 ± 9.82				
SARS-CoV-2	MMIP	43.50 ± 2.76	74.33 ± 4.72	95.61 ± 9.54	206.73 ± 24.47	5.48	3.50	1.49	1.27
	MNIP	7.94 ± 1.19	21.22 ± 1.69	63.75 ± 6.37	162.75 ± 16.22				

## Supplementary Materials

The following are available online at https://www.mdpi.com/article/10.3390/nano11112985/s1, Figure S1: Protein complex representing interactions between receptor binding domain (RBD) of the spike protein specific to the SARS-CoV-2 with the ACE 2 receptor (PDB ID: 6M0J). The highlighted sequence (green) was chosen as the target template/epitope for imprinting. Figure S2: TEM micrographs of the prepared MNPs. Figure S3: Photographs of the MNIPs/MMIPs with different amounts of peptides and prepared with different polymerization times. A) Nº1 is MNIP-1, Nº2 is MMIP-05-1, Nº3 is MMIP-1-1, Nº4 is MMIP-2-1, Nº5 is MMIP-3-1 and Nº6 is MMIP-4-1. B) Nº1 is MNIP-2, Nº2 is MMIP-05-2, Nº3 is MMIP-1-2, Nº4 is MMIP-2-2, Nº5 is MMIP-3-2 and Nº6 is MMIP-4-2. C) Nº1 is MNIP-24, Nº2 is MMIP-05-24, Nº3 is MMIP-1-24, Nº4 is MMIP-2-24, Nº5 is MMIP-3-24 and Nº6 is MMIP-4-24. Figure S4: Photographs of the dispersion of A) MNPs and B) MMIP/MNIP. Figure S5: Photographs of A) dopamine and B) polydopamine solutions. Figure S6: Representation of nine docked poses of dopamine in the binding energy range of A) −3.2 to −3.4 kcal/mol with SARS-CoV-2 peptide and B) −3.6 to −4.0 kcal/mol with Zika virus peptide.
