# Supplementary material for: Development and Characterization of Magnetic SARS-CoV-2 Peptide-Imprinted Polymers
Source: Nanomaterials (Basel). 2021 Nov 6;11(11):2985. doi: 10.3390/nano11112985 (PMC8618860; doi:10.3390/nano11112985)
Supplement: Supplementary file 1 [file nanomaterials-11-02985-s001.zip › nanomaterials-1429334-supplementary.pdf]

# Development and characterization of magnetic SARS-CoV-2 peptide−imprinted polymers

Beatriz Fresco−Cala [1,*], Soumya Rajpal [1,2], Tamara Rudolf [1], Benedikt Keitel [1], Rüdiger Groß [3], Jan Münch [3], Alex D. Batista [1,*] and Boris Mizaikoff [1,4].

[1]Institute of Analytical and Bioanalytical Chemistry, Ulm University, 89081 Ulm, Germany; beatriz.fresco−cala@uni−ulm.de (B.F.−C.); rajpalsoumya@yahoo.com (S.R.); tamara.rudolf@uni−ulm.de (T.R.); benedikt.keitel@uni−ulm.de (B.K.); alexbatista@ufu.br (A.D.B.); boris.mizaikoff@uni−ulm.de (B.M.)

[2]Department of Biochemical Engineering and Biotechnology, Indian Institute of Technology Delhi, 110016, New Delhi, India; rajpalsoumya@yahoo.com (S.R.)

[3]Institute of Molecular Virology, Ulm University Medical Center, 89081 Ulm, Germany; boris.mizaikoff@uni−ulm.de (B.M.)

[4]Hahn−Schickard, 89077 Ulm, Germany; ruediger.gross@uni−ulm.de (R.G.); jan.muench@uni−ulm.de (J.M.)

[*]Correspondence: beatriz.fresco−cala@uni−ulm.de (B.F.−C.); alexbatista@ufu.br (A.D.B.)

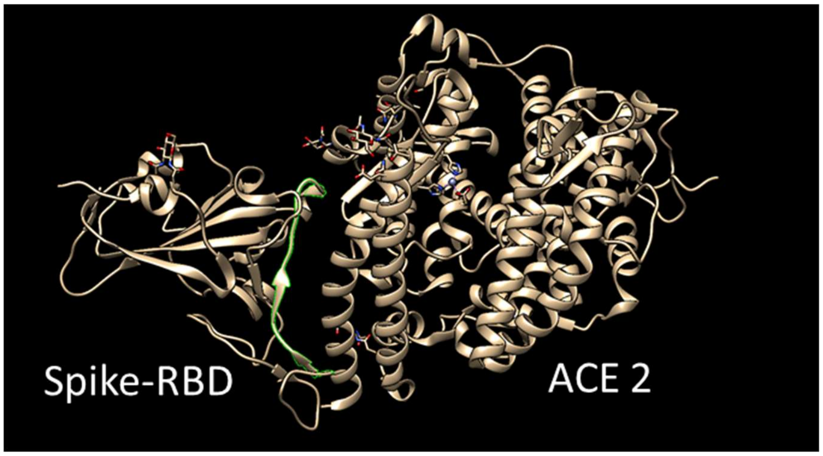


**Figure S1.** Protein complex representing interactions between receptor binding domain (RBD) of the spike protein specific to the SARS-CoV-2 with the ACE 2 receptor (PDB ID: 6M0J). The highlighted sequence (green) was chosen as the target template/epitope for imprinting.

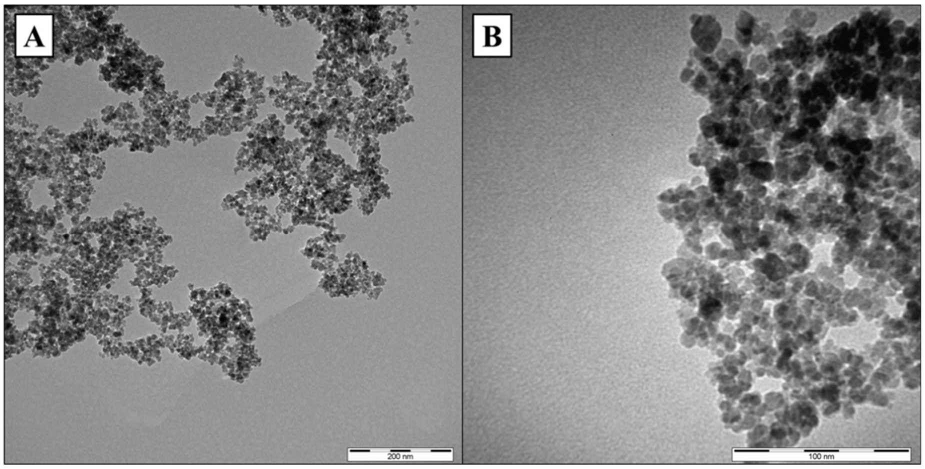


**Figure S2.** TEM micrographs of the prepared MNPs.

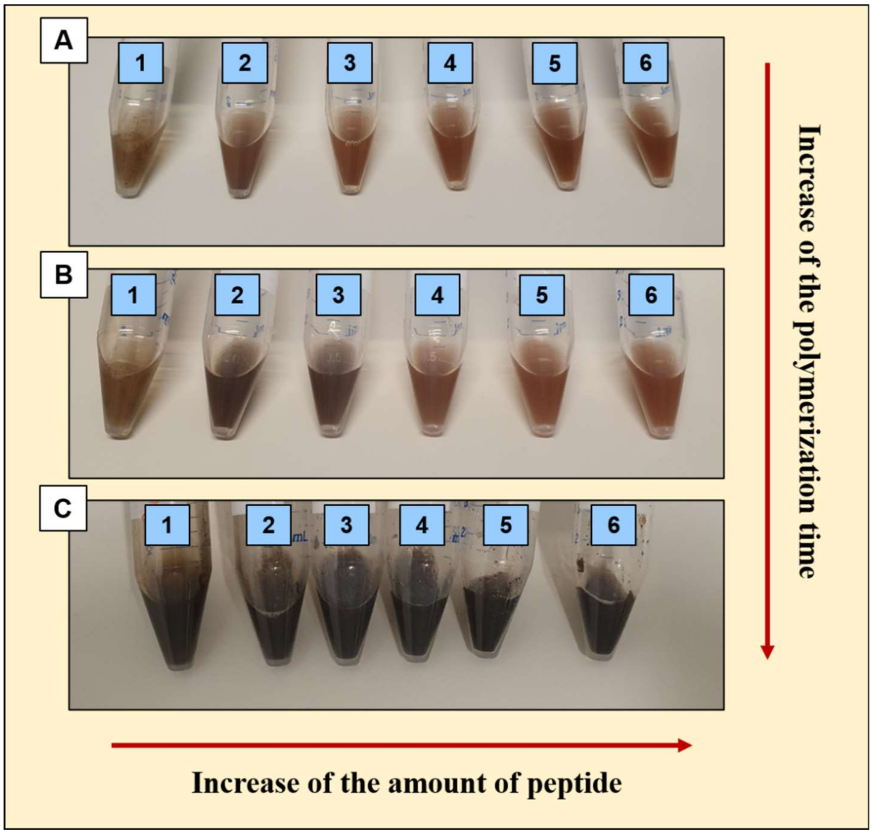


**Figure S3.** Photographs of the MNIPs/MMIPs with different amounts of peptides and prepared with different polymerization times. **A)** Nº1 is MNIP-1, Nº2 is MMIP-05-1, Nº3 is MMIP-1-1, Nº4 is MMIP-2-1, Nº5 is MMIP-3-1 and Nº6 is MMIP-4-1. **B)** Nº1 is MNIP-2, Nº2 is MMIP-05-2, Nº3 is MMIP-1-2, Nº4 is MMIP-2-2, Nº5 is MMIP-3-2 and Nº6 is MMIP-4-2. **C)** Nº1 is MNIP-24, Nº2 is MMIP-05-24, Nº3 is MMIP-1-24, Nº4 is MMIP-2-24, Nº5 is MMIP-3-24 and Nº6 is MMIP-4-24.

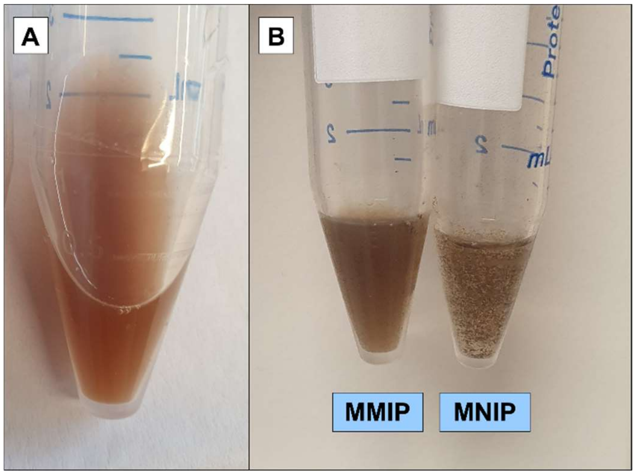


**Figure S4.** Photographs of the dispersion of **A)** MNPs and **B)** MMIP/MNIP.

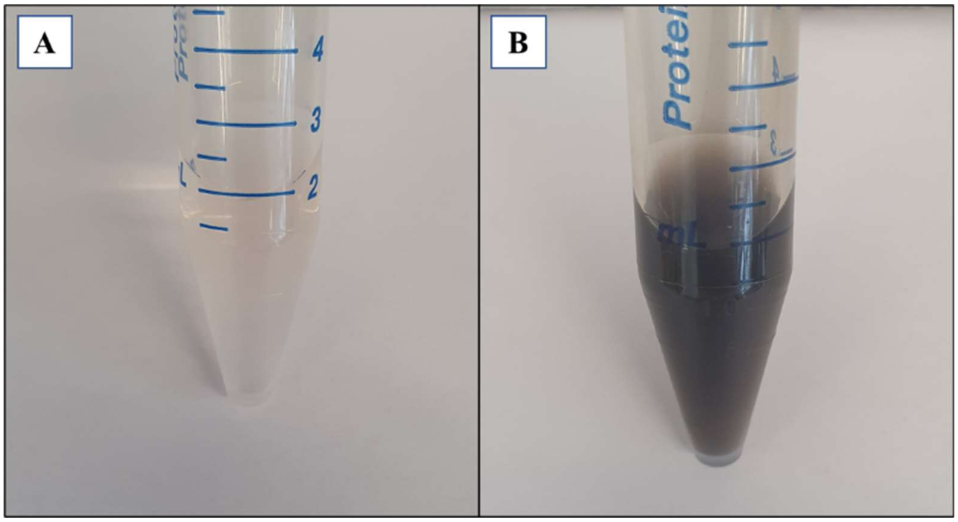


**Figure S5.** Photographs of **A)** dopamine and **B)** polydopamine solutions.

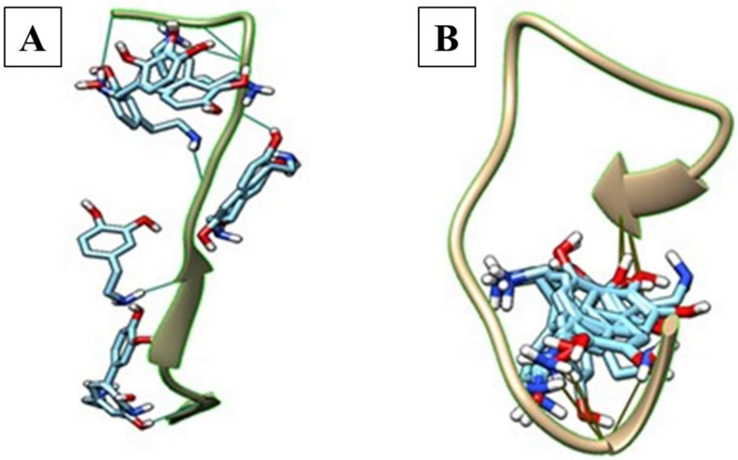


**Figure S6.** Representation of nine docked poses of dopamine in the binding energy range of **A)** -3.2 to -3.4 kcal/mol with SARS-CoV-2 peptide and **B)** -3.6 to -4.0 kcal/mol with Zika virus peptide.
